# Supplementary material for: Effects of Exercise Training on Peripheral Muscle Strength in Children and Adolescents with Cystic Fibrosis: A Meta-Analysis
Source: Healthcare (Basel). 2022 Dec 13;10(12):2520. doi: 10.3390/healthcare10122520 (PMC9778003; doi:10.3390/healthcare10122520)
Supplement: Supplementary file 1 [file healthcare-10-02520-s001.zip › File S1 Full search strategy.pdf]

File S1. Full search strategy

Keywords used for literature search are detailed below:

Condition	Cystic fibrosis
**AND**	
Population	Child ; children ; adolescent ; adolescence ; teenager ; teen ; youth ; school age
**AND**	
Intervention	Physical activity ; exercise ; exercise training ; physical exercise ; exercise program ; strength training ; strengthening program ; resistance training ; weight-lifting ; weight-bearing ; aerobic training; endurance training; anaerobic ; interval training ; running ; run ; jogging ; biking ; bike

Research algorithms for each database were as follows, according to the 2020 PRISMA guidelines:

For research on Pubmed, the complete search was:

((“cystic fibrosis [All fields]” OR “cystic fibrosis [mesh]”) AND (“child [All fields]” OR “children [All fields]” OR “adolescent [All fields]” OR “adolescence [All fields]” OR “teenager [All fields]” OR “teen [All fields]” OR “youth [All fields]” OR “school age [All fields]”) AND (“physical activity [All fields]” OR “exercise [All fields]” OR “exercise [mesh]” OR “exercise training [All fields]” OR “physical exercise [All fields]” OR “exercise program [All fields]” OR “strength training [All fields]” OR “strengthening program [All fields]” OR “resistance training [All fields]” OR “resistance training [mesh]” OR “weight-lifting [All fields]” OR “weight-bearing [All fields]” OR “aerobic training [All fields]” OR “endurance training [mesh]” OR “anaerobic [All fields]” OR “interval training [All fields]” OR “running [All fields]” OR “run [All fields]” OR “jogging [All fields]” OR “biking [All fields]” OR “bike [All fields]”))

For research on ScienceDirect and CENTRAL, the complete search was:

(child OR children OR adolescent) AND (cystic fibrosis) AND (physical activity OR physical exercise OR exercise)

The filter “research articles” was used on ScienceDirect.

For research on PEDro, the complete search was:

Cystic fibrosis (Abstract & Title), Fitness training (Therapy), chronic respiratory disease (Topic)

For research on OpenGrey, and in the databases of the ATS, ERS and ECFS congresses, the complete search was:

(child OR children OR adolescent) AND (cystic fibrosis) AND (physical activity OR physical exercise OR “exercise)
