# Supplementary material for: Effects of Exercise Training on Peripheral Muscle Strength in Children and Adolescents with Cystic Fibrosis: A Meta-Analysis
Source: Healthcare (Basel). 2022 Dec 13;10(12):2520. doi: 10.3390/healthcare10122520 (PMC9778003; doi:10.3390/healthcare10122520)
Supplement: Supplementary file 1 [file healthcare-10-02520-s001.zip › File S2 Full-text studies rejected.pdf]

File S2

Table S1. Full-text studies rejected with reasons

Studies that did not meet inclusion criteria are listed below.

Studies	Reasons for non-inclusion
Schneiderman-Walker *et al*., 2000 (1)	Not a physical exercise intervention
Salonini *et al*., 2015 (2)	Not investigating the outcomes of this review
Boucher *et al*., 1997 (3)	Not a physical exercise intervention
Dassios *et al*., 2013 (4)	Not a physical exercise intervention
Pérez *et al*., 2014 (5)	Not a physical exercise intervention
Franco *et al*., 2014 (6)	Population did not fulfill the inclusion criteria (adults and children)
Klijn *et al*., 2003 (7)	Non-controlled trial
Pfirrmann *et al*., 2018 (8)	Population did not fulfill the inclusion criteria (adults only)
Dietz-Terjung *et al*., 2020 (9)	Population did not fulfill the inclusion criteria (adults and children)
Baker *et al*., 2006 (10)	Non-controlled trial
Van de Weert-Van Leeuwan *et al*., 2012 (11)	Non-controlled trial
Zach *et al*., 1982 (12)	Non-controlled trial
Stanghelle *et al*., 1988 (13)	Non-controlled trial
Zach *et al*., 1981 (14)	Non-controlled trial

Gruber *et al*., 2020 (15)	Non-controlled trial
Urquhart *et al*., 2012 (16)	Non-controlled trial
Edlund *et al*., 1986 (17)	Non-controlled trial
Hommerding *et al*., 2015 (18)	Population did not fulfill the inclusion criteria (children until the age of 21 years old); not a physical exercise intervention
Elbasan *et al*., 2012 (19)	Non-controlled trial
Schmidt *et al*., 2011 (20)	Population did not fulfill the inclusion criteria (adults only)
Paranjape *et al*., 2012 (21)	Non-controlled trial
Cropp *et al*., 1982 (22)	Not investigating the outcomes of this review
Gruber *et al*., 2008 (23)	Non-controlled trial
Andréasson *et al*., 1987 (24)	Non-controlled trial
Blomquist *et al*., 1986 (25)	Non-controlled trial
Cerny *et al*., 1989 (26)	Not investigating the outcomes of this review
Orenstein *et al.,* 2004 (27)	Non-controlled trial
Chen *et al.,* 2018 (28)	Non-controlled trial

References:

1. Schneiderman-Walker J, Pollock SL, Corey M, Wilkes DD, Canny GJ, Pedder L, et al. A randomized controlled trial of a 3-year home exercise program in cystic fibrosis. J Pediatr. 2000 Mar;136(3):304–10.

2. Salonini E, Gambazza S, Meneghelli I, Tridello G, Sanguanini M, Cazzarolli C, et al. Active Video Game Playing in Children and Adolescents With Cystic Fibrosis: Exercise or Just Fun? Respiratory Care. 2015 Aug 1;60(8):1172–9.

3. Boucher GP, Lands LC, Hay JA, Hornby L. Activity levels and the relationship to lung function and nutritional status in children with cystic fibrosis. Am J Phys Med Rehabil. 1997 Aug;76(4):311–5.

4. Dassios T, Katelari A, Doudounakis S, Dimitriou G. Aerobic exercise and respiratory muscle strength in patients with cystic fibrosis. Respir Med. 2013 May;107(5):684–90.

5. Pérez M, Groeneveld IF, Santana-Sosa E, Fiuza-Luces C, Gonzalez-Saiz L, Villa-Asensi JR, et al. Aerobic fitness is associated with lower risk of hospitalization in children with cystic fibrosis. Pediatr Pulmonol. 2014 Jul;49(7):641–9.

6. Franco CB, Ribeiro AF, Morcillo AM, Zambon MP, Almeida MB, Rozov T. Air stacking: effects of Pilates mat exercises on muscle strength and on pulmonary function in patients with cystic fibrosis. J Bras Pneumol. 2014 Oct;40(5):521–7.

7. Klijn PH, Terheggen-Lagro SW, Van Der Ent CK, Van Der Net J, Kimpen JL, Helders PJ. Anaerobic exercise in pediatric cystic fibrosis. Pediatr Pulmonol. 2003 Sep;36(3):223–9.

8. Pfirrmann D, Haller N, Huber Y, Jung P, Lieb K, Gockel I, et al. Applicability of a Web-Based, Individualized Exercise Intervention in Patients With Liver Disease, Cystic Fibrosis, Esophageal Cancer, and Psychiatric Disorders: Process Evaluation of 4 Ongoing Clinical Trials. JMIR Res Protoc. 2018 May 22;7(5):e106.

9. Dietz-Terjung S, Gruber W, Sutharsan S, Taube C, Olivier M, Mellies U, et al. Association between habitual physical activity (HPA) and sleep quality in patients with cystic fibrosis. Sleep Breath. 2020 Jul 14;

10. Baker CF, Wideman L. Attitudes toward physical activity in adolescents with cystic fibrosis: sex differences after training: a pilot study. J Pediatr Nurs. 2006 Jun;21(3):197–210.

11. van de Weert-van Leeuwen PB, Slieker MG, Hulzebos HJ, Kruitwagen CLJJ, van der Ent CK, Arets HGM. Chronic infection and inflammation affect exercise capacity in cystic fibrosis. Eur Respir J. 2012 Apr;39(4):893–8.

12. Zach M, Oberwaldner B, Häusler F. Cystic fibrosis: physical exercise versus chest physiotherapy. Arch Dis Child. 1982 Aug;57(8):587–9.

13. Stanghelle JK, Hjeltnes N, Bangstad HJ, Michalsen H. Effect of daily short bouts of trampoline exercise during 8 weeks on the pulmonary function and the maximal

oxygen uptake of children with cystic fibrosis. Int J Sports Med. 1988 Feb;9 Suppl 1:32–6.

14. Zach MS, Purrer B, Oberwaldner B. Effect of swimming on forced expiration and sputum clearance in cystic fibrosis. Lancet. 1981 Nov 28;2(8257):1201–3.

15. Gruber W, Stehling F, Olivier M, Dillenhoefer S, Koerner-Rettberg C, Sutharsan S, et al. Effects of a long-term exercise program on motor performance in children and adolescents with CF. Pediatr Pulmonol. 2020 Dec;55(12):3371–80.

16. Urquhart D, Sell Z, Dhouieb E, Bell G, Oliver S, Black R, et al. Effects of a supervised, outpatient exercise and physiotherapy programme in children with cystic fibrosis. Pediatr Pulmonol. 2012 Dec;47(12):1235–41.

17. Edlund LD, French RW, Herbst JJ, Ruttenburg HD, Ruhling RO, Adams TD. Effects of a swimming program on children with cystic fibrosis. Am J Dis Child. 1986 Jan;140(1):80–3.

18. Hommerding PX, Baptista RR, Makarewicz GT, Schindel CS, Donadio MVF, Pinto LA, et al. Effects of an educational intervention of physical activity for children and adolescents with cystic fibrosis: a randomized controlled trial. Respir Care. 2015 Jan;60(1):81–7.

19. Elbasan B, Tunali N, Duzgun I, Ozcelik U. Effects of chest physiotherapy and aerobic exercise training on physical fitness in young children with cystic fibrosis. Ital J Pediatr. 2012 Jan 10;38:2.

20. Schmidt AM, Jacobsen U, Bregnballe V, Olesen HV, Ingemann-Hansen T, Thastum M, et al. Exercise and quality of life in patients with cystic fibrosis: A 12-week intervention study. Physiother Theory Pract. 2011 Nov;27(8):548–56.

21. Paranjape SM, Barnes LA, Carson KA, von Berg K, Loosen H, Mogayzel PJ. Exercise improves lung function and habitual activity in children with cystic fibrosis. J Cyst Fibros. 2012 Jan;11(1):18–23.

22. Cropp GJ, Pullano TP, Cerny FJ, Nathanson IT. Exercise tolerance and cardiorespiratory adjustments at peak work capacity in cystic fibrosis. Am Rev Respir Dis. 1982 Aug;126(2):211–6.

23. Gruber W, Orenstein DM, Braumann KM, Hüls G. Health-related fitness and trainability in children with cystic fibrosis. Pediatr Pulmonol. 2008 Oct;43(10):953–64.

24. Andréasson B, Jonson B, Kornfält R, Nordmark E, Sandström S. Long-term effects of physical exercise on working capacity and pulmonary function in cystic fibrosis. Acta Paediatr Scand. 1987 Jan;76(1):70–5.

25. Blomquist M, Freyschuss U, Wiman LG, Strandvik B. Physical activity and self treatment in cystic fibrosis. Arch Dis Child. 1986 Apr;61(4):362–7.

26. Cerny FJ. Relative effects of bronchial drainage and exercise for in-hospital care of patients with cystic fibrosis. Phys Ther. 1989 Aug;69(8):633–9.

27. Orenstein DM, Hovell MF, Mulvihill M, Keating KK, Hofstetter CR, Kelsey S, et al. Strength vs aerobic training in children with cystic fibrosis: a randomized controlled trial. Chest. 2004 Oct;126(4):1204–14.

28. Chen JJ, Cooper DM, Haddad F, Sladkey A, Nussbaum E, Radom-Aizik S. Tele-Exercise as a Promising Tool to Promote Exercise in Children With Cystic Fibrosis. Front Public Health. 2018;6:269.
