# Supplementary material for: Effects of Exercise Training on Peripheral Muscle Strength in Children and Adolescents with Cystic Fibrosis: A Meta-Analysis
Source: Healthcare (Basel). 2022 Dec 13;10(12):2520. doi: 10.3390/healthcare10122520 (PMC9778003; doi:10.3390/healthcare10122520)
Supplement: Supplementary file 1 [file healthcare-10-02520-s001.zip › File S3 Funnel plots.pdf]

File S3. Funnel plots and Egger's bias statistics regression

Figure S1. Lower limb muscle strength

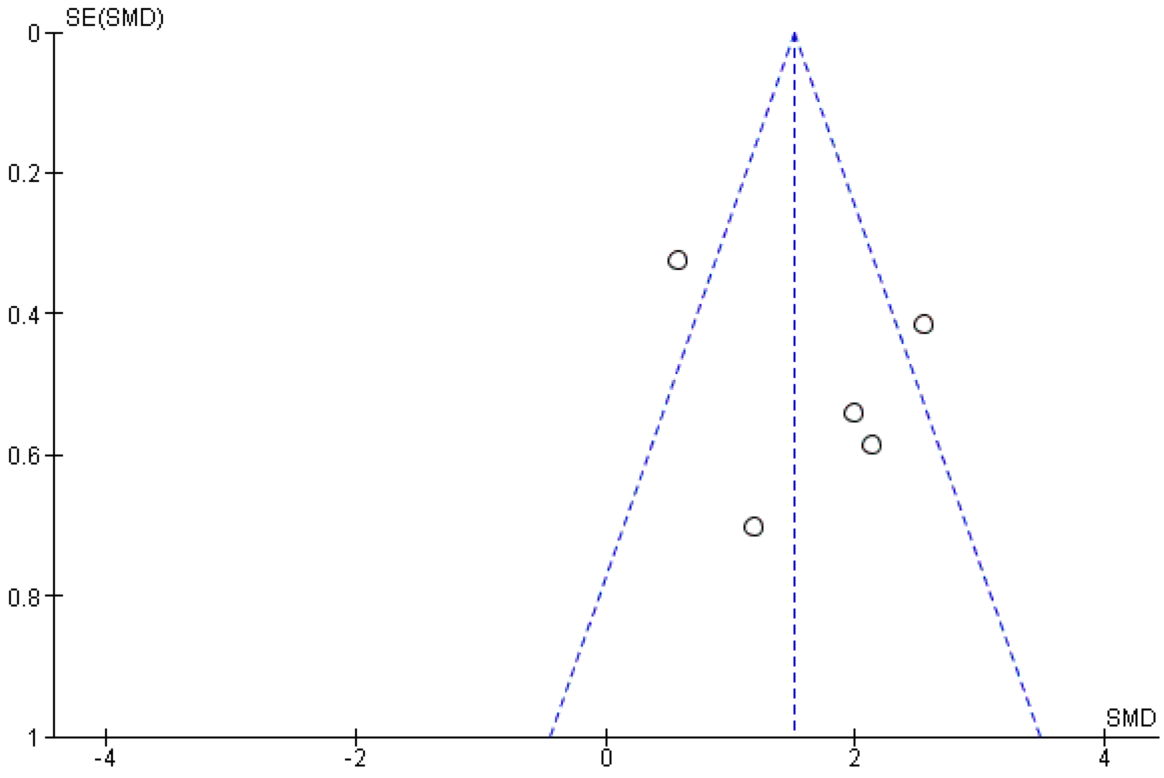

Egger Regression				
	Estimate	Standard error	CI LL	CI UL
Intercept	1,84	6,84	-17,15	20,82
Slope	-0,12	6,66	-18,61	18,38
t test	0,27			
p-value	0,805			

Figure S2. Upper limb muscle strength

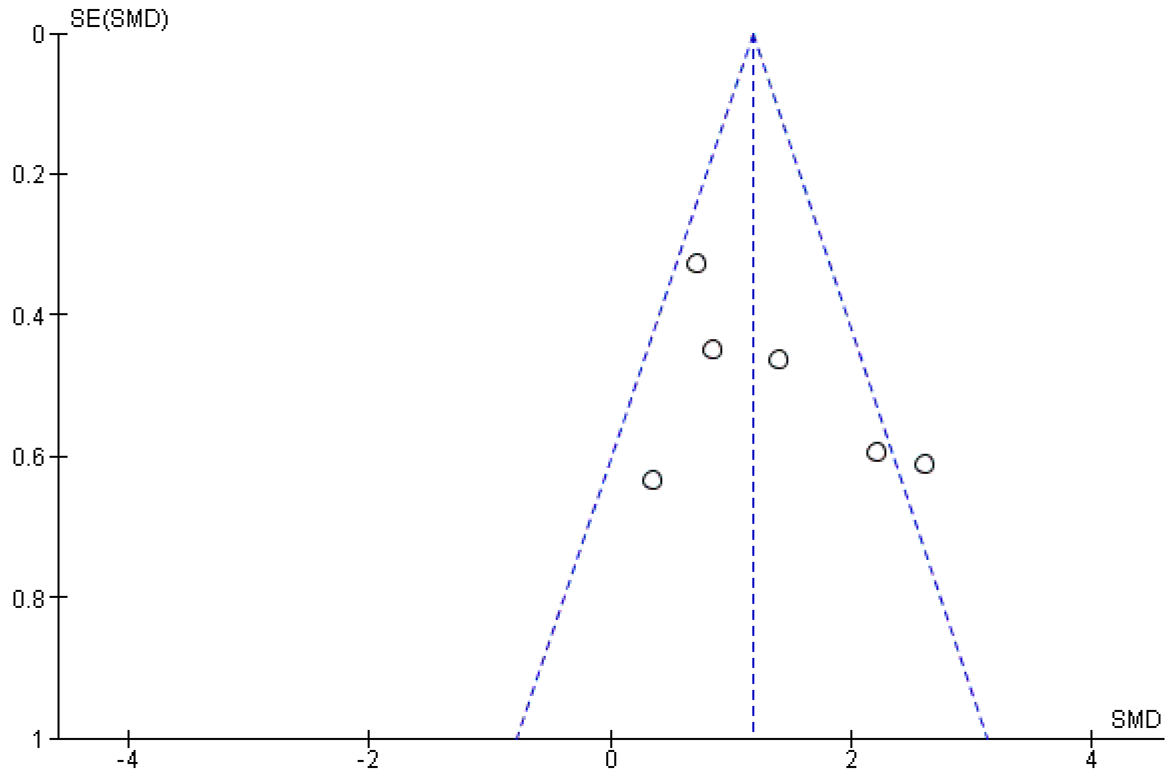

Egger Regression				
	Estimate	Standard error	CI LL	CI UL
Intercept	4,81	5,77	-10,03	19,65
Slope	-2,56	4,66	-14,52	9,41
t test	0,83			
p-value	0,451			

Figure S3. Muscle mass

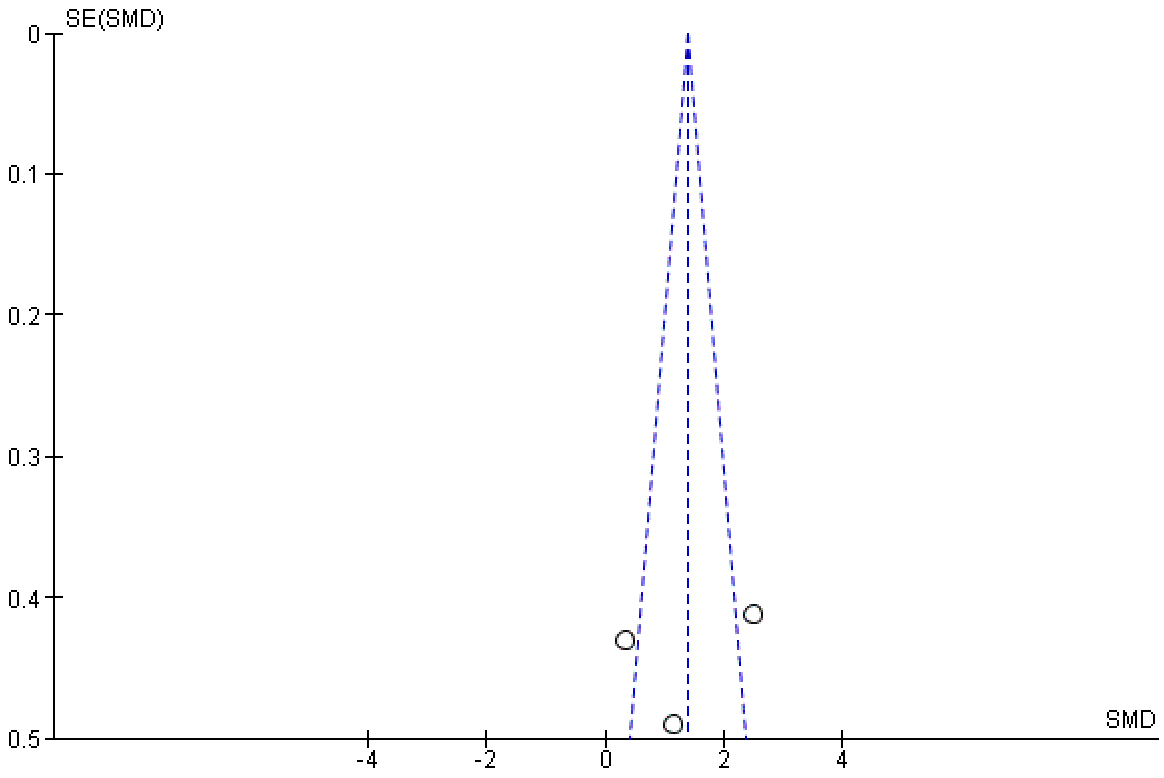

Egger Regression				
	Estimate	Standard error	CI LL	CI UL
Intercept	-33,01	64,58	-	-
Slope	39,62	74,92	-	-
t test	-0,51			
p-value	0,699			

Figure S4. VO_2peak

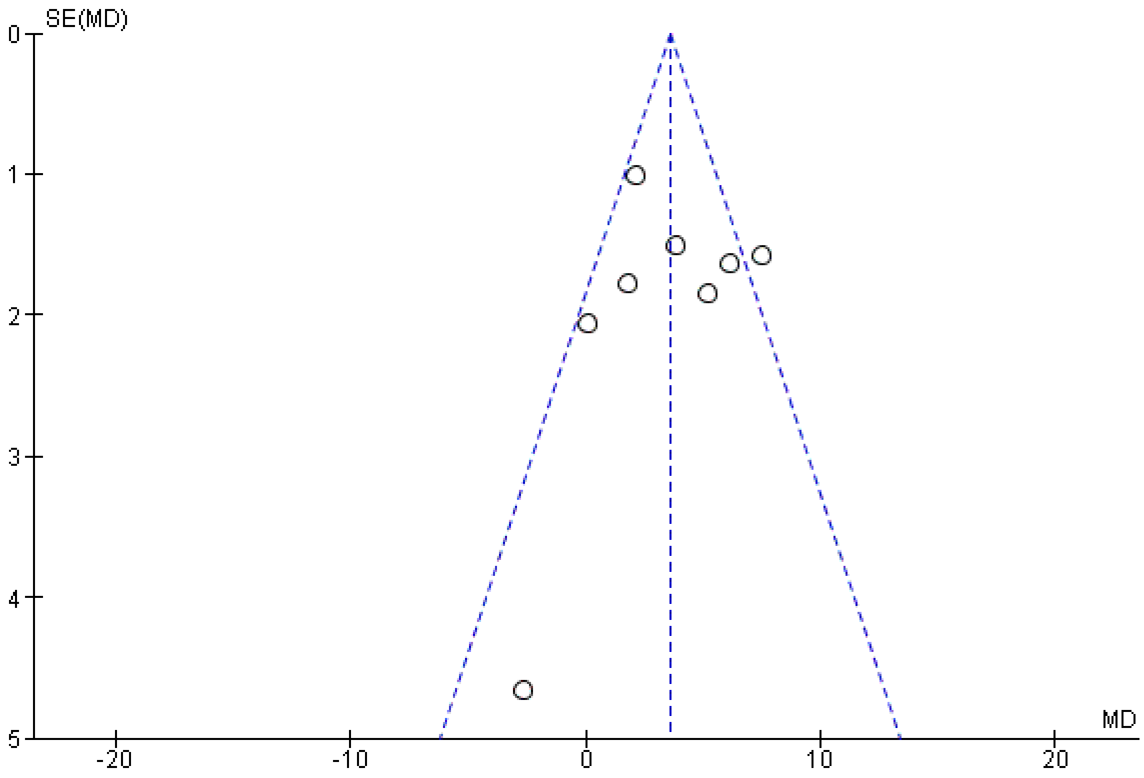

Egger Regression				
	Estimate	Standard error	CI LL	CI UL
Intercept	5,72	3,62	-2,84	14,28
Slope	-2,88	2,26	-8,22	2,46
t test	1,58			
p-value	0,165			

Figure S5. Physical activity level

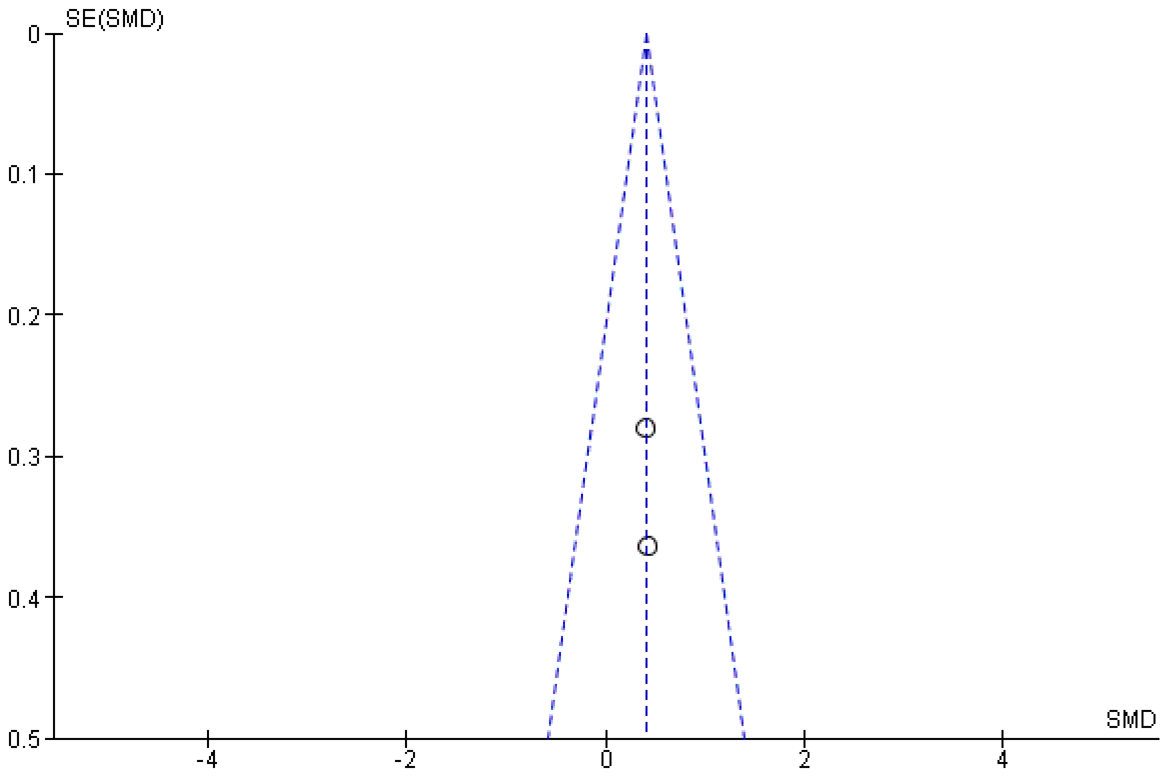

**Egger Regression : impossible to compute due to the lack of data**
