# Supplementary material for: Effects of Exercise Training on Peripheral Muscle Strength in Children and Adolescents with Cystic Fibrosis: A Meta-Analysis
Source: Healthcare (Basel). 2022 Dec 13;10(12):2520. doi: 10.3390/healthcare10122520 (PMC9778003; doi:10.3390/healthcare10122520)
Supplement: Supplementary file 1 [file healthcare-10-02520-s001.zip › File S4 Exercise interventions.pdf]

File S4

Table S2. Detailed exercise training interventions

**Selvadurai *et al.* (2002)**	**I.1:** Aerobic exercise (treadmill running / cycle ergometer) supervised, 30min per session, 70% maximal HR, 5 sessions/week, 1 week<br>**I.2:** ULM and LLM strengthening supervised, non-isokinetic resistance machine, 70% maximal subjective resistance, 5 sessions/week, 1 week<br>**C:** Standard chest physiotherapy
**Klijn *et al.* (2004)**	**I:** Anaerobic exercise supervised (children's own physiotherapist on the basis of a booklet), circuit training: (i.e., 10x5m sprint, shifting balls from point A to point B, basketball/soccer, relay race, hopscotch, jumping obstacles, jump, hop, running sideways and backwards), 3 repetitions of five 30-seconds periods separated by a 1-minute break per exercise), 2 sessions/week, 12 weeks<br>**C:** Habitual CF care
**Luke-Zeitoun *et al.* (2012)**	**I:** Individualized exercise (personal trainer) non-supervised, weekly video conference for readjustments, 6 months<br>**C:** Habitual CF care

**Santana Sosa *et al.* (2012)**	**I:** Aerobic exercise : supervised, cycle ergometer, 10min warm-up, 20 to 40min per session, HR at the ventilatory threshold; Strength exercise : 3 circuits of 11 exercises without rest period (12-15rep/exercise, 40 to 60% of 5RM) (*bench press, shoulder press, leg extension, leg press, leg curl, abdominal crunch, low back extension, arm curl, elbow extension, seated row, lateral pulldown*), 3 sessions/week, 8 weeks<br>**C:** Habitual CF care + PA information
**Santana Sosa *et al.* (2014)**	**I:** Aerobic exercise: supervised, cycle ergometer, 10min warm-up, 20 to 40min per session, HR at the ventilatory threshold + 15min active playing (running/soccer playing); Strength exercise: 3 circuits of 11 strength exercises without rest periods (12-15rep/exercise, beginning 50% of 5RM, +2.25kg every 3 sessions) (*bench press, shoulder press, leg extension, leg press, leg curl, abdominal crunch, low back extension, arm curl, elbow extension, seated row, lateral pulldown*), 3 sessions/week, 8 weeks; IMT: 2/day, 5min/session (30 inspirations, 40-50% of MIP for 4 weeks, then 40% of the new MIP measured at 4 weeks)<br>**C:** Habitual CF care + PA information + Sham IMT (same protocol, 10% of MIP)
**Ledger *et al.* (2016)**	**I**: Individualized exercise supervised (aerobic, strength and core), 1 session/week, 24 months<br>**C:** Habitual CF care
**Del Corral *et al.* (2018)**	**I:** Home-based active video game (Nintendo Wii™) exercise supervised (parents/caregivers), running/squat/lunges/bicep curls, 30 to 60min per session, 70 to 80% of maximal HR, workload increased every week, 5 sessions/week, 6 weeks<br>**C:** Habitual CF care

**Gupta *et al.* (2019)**	**I:** Strength home-based exercise, squats/forward lunges/raise-heel drops/push-ups/quadriceps strengthening, plyometric jumps (20/day), workload increased every 3 months, 3 sessions/week, 1 year + vitamin D and calcium supplementation<br><br>**C:** Habitual CF care + vitamin D and calcium supplementation
**Estevez-Gonzalez *et al.* (2021)**	**I:** Supervised (1:1 ratio) strength exercise (ULM, LLM and core), 60-min sessions: warm-up (15 min), resistance exercises (35 min) and cool-down (10 min), bilateral seated row/seated bench press/leg press/leg extension/leg flexion on pediatric machines, (i) initially 60–70% of 1RM, 8–12 repetitions, 3 series for 2 weeks; (ii) finally 70–80% of 1RM, 6–8 repetitions, 3 series for 6 weeks; 3 sessions/week, 8 weeks<br><br>**C:** Habitual CF Care
**Donadio *et al.* (2022)**	**I.1:** Supervised strength exercise (ULM, LLM and core), 60-min sessions: warm-up (15 min), resistance training (35 min) and cool down (10 min), 6 exercises: bench press/leg extension/leg press/leg curl/seated row/chest pull, beginning 12–15 repetitions, ending 8–10 repetitions (60s rest period between sets and 2 min between exercises), from 40-60% of the 1RM to 70–80%, 3 sessions/week, 8 weeks<br><br>**I.2:** Same strength training protocol + NMES for the quadriceps and interscapular region simultaneously, 4 electrodes (2: 8 × 4 cm and 2: 4 × 4 cm for each muscle group), 250ms pulse, 4 Hz frequency, and duration of 2s on (concentric) and 4s off (eccentric), individualized intensity<br><br>**C:** Habitual CF care

Abbreviation: C: control group; CF: cystic fibrosis; HR: heart rate; Hz: hertz; I: intervention group; IMT: inspiratory muscle training; LLM: lower limb muscles; MIP: maximal inspiratory pressure; NMES: neuromuscular electrical stimulation; PA: physical activity; ULM: upper limb muscles; VT: ventilatory threshold; 1RM: one-repetition maximal resistance; 5RM: five-repetition maximal resistance
