# Supplementary material for: Effects of Exercise Training on Peripheral Muscle Strength in Children and Adolescents with Cystic Fibrosis: A Meta-Analysis
Source: Healthcare (Basel). 2022 Dec 13;10(12):2520. doi: 10.3390/healthcare10122520 (PMC9778003; doi:10.3390/healthcare10122520)
Supplement: Supplementary file 1 [file healthcare-10-02520-s001.zip › File S5 Methods of muscle strength measurement.pdf]

File S5.

Table S3. Detailed methods for muscle strength measurement

Study details	Muscle strength measurement
Selvadurai *et al.* (2002)	LLM: isokinetic dynamometry for quadriceps and hamstrings of the nondominant leg, 3-min rest before measure, best of three measurements
Klijn *et al.* (2004)	LLM and ULM: isometric handheld dynamometry 4 muscle groups (shoulder abductors, elbow flexors, hip extensors and knee extensors), total maximal muscle force in the four muscle groups
Luke-Zeitoun *et al.* (2012)	LLM: Sits-up, ULM: push-up *(no other information provided)*
Santana Sosa *et al.* (2012)	LLM and ULM: seated bench/seated row/seated leg press measures of 5RM, 3 warm-ups (50, 70 and 90% of the perceived 5RM) separated by 1-min rest, 2-mins rest and then maximum of 2 attempts/day for 5RM measurement (starting at 100-105% of the perceived 5RM)
Santana Sosa *et al.* (2014)	LLM and ULM: seated bench/seated row/seated leg press measures of 5RM, 3 warm-ups (50, 70 and 90% of the perceived 5RM) separated by 1-min rest, 2-mins rest and then maximum of 2 attempts/day for 5RM measurement (starting at 100-105% of the perceived 5RM)
Del Corral *et al.* (2018)	LLM: Horizontal jump test, feet placed at shoulder width, 3 jumps, farthest distance recorded<br>ULM: Medicine ball throw, participants on their knees throwing the ball forward (overhead motion) (2 kg: ≤ 12 years of age and 3 kg: ≥ 13 years of age), 3 throws, farthest distance recorded

Estevez-Gonzalez *et al.* (2021)	LLM and ULM: leg press/seated bench press/seated bilateral row measures of 5RM, 3 warm-ups (50, 70, and 90% of the perceived 5RM) separated by 1min of rest, 2- to 3-min resting period, 5RM attempt (100% of the perceived effort)
Donadio *et al.* (2022)	LLM and ULM: low paddle/pull ahead/chest press/leg press/knee extension/knee flexion measures of 5RM, 2 warm-ups (50 and 70% of the perceived 5RM) separated by 1min of rest, 2 min of resting, 5RM attempt (90-105% of 5RM), increase 2.5–5% for a new attempt if needed (2-min rest); maximum 3 attempts/day

Abbreviation: C: control group; HR: heart rate; I: intervention group; IMT: inspiratory muscle training; LLM: lower limb muscles; ULM: upper limb muscles; VT: ventilatory threshold; 1RM: one-repetition maximal resistance; 5RM: five-repetition maximal resistance
