# Supplementary material for: Effects of Exercise Training on Peripheral Muscle Strength in Children and Adolescents with Cystic Fibrosis: A Meta-Analysis
Source: Healthcare (Basel). 2022 Dec 13;10(12):2520. doi: 10.3390/healthcare10122520 (PMC9778003; doi:10.3390/healthcare10122520)

File S6. Forest plot of comparison and risk of bias for the secondary outcomes

Figure S6. Muscle mass

Study or Subgroup	Exercise Mean	Exercise SD	Exercise Total	Control Mean	Control SD	Control Total	Weight	Std. mean difference IV, Random, 95% CI	A	B	C	D	E	F
Santana-Sosa et al. 2012	1.3	6.2	11	-0.2	1	11	33.6%	0.32 [-0.52 , 1.17]	-	+	+	+	!	-
Santana-Sosa et al. 2014	1	1.3	10	-0.1	0.2	10	32.3%	1.13 [0.17 , 2.09]	-	+	+	+	!	-
Selvadurai et al. 2002	1.5	0.4	22	0.6	0.3	22	34.1%	2.50 [1.69 , 3.31]	+	!	+	!	!	!
**Total (95% CI)**			**43**			**43**	**100.0%**	**1.33 [0.02 , 2.64]**						

Heterogeneity: Tau² = 1.14; Chi² = 13.70, df = 2 (P = 0.001); I² = 85%
Test for overall effect: Z = 1.98 (P = 0.05)
Test for subgroup differences: Not applicable

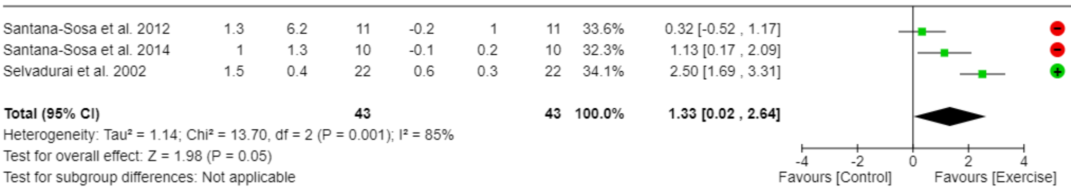

Risk of bias legend
(A) Randomisation process
(B) Deviations from the intended interventions
(C) Missing outcome data
(D) Measurement of the outcome
(E) Selection of the reported result
(F) Overall bias

+ Low risk
! Some concerns
- High risk

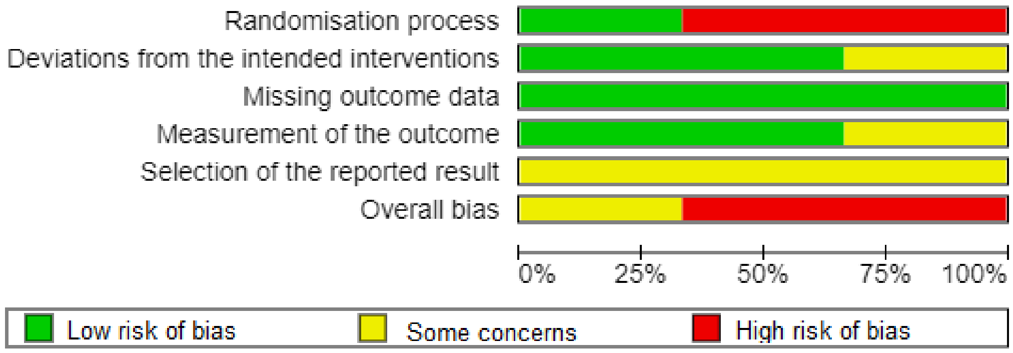

Figure S7. VO_2peak

Study or Subgroup	Exercise Mean	Exercise SD	Exercise Total	Control Mean	Control SD	Control Total	Weight	Mean difference IV, Random, 95% CI	A	B	C	D	E	F
Estevez-Gonzalez et al. 2021	-2.8	11.7	12	-0.2	11.1	12	3.5%	-2.60 [-11.72 , 6.52]	-	-	-	+	+	-
Donadio et al. 2022	-0.2	2.6	11	-0.3	6.3	11	11.1%	0.10 [-3.93 , 4.13]	-	-	-	+	+	-
Ledger et al. 2016	7.4	7.5	37	5.6	7.5	34	12.7%	1.80 [-1.69 , 5.29]	!	-	+	-	!	-
Klijn et al. 2004	1.5	2.6	11	-0.6	1.9	9	18.2%	2.10 [0.12 , 4.08]	+	-	-	+	!	-
Gupta et al. 2019	2.4	4.8	25	-1.4	6	27	14.6%	3.80 [0.86 , 6.74]	+	+	+	+	+	+
Selvadurai et al. 2002	4	6.1	22	-1.2	6.2	22	12.2%	5.20 [1.57 , 8.83]	+	!	+	-	!	-
Santana-Sosa et al. 2012	3.9	2.9	11	-2.2	4.6	11	13.6%	6.10 [2.89 , 9.31]	-	+	+	+	!	-
Santana-Sosa et al. 2014	6.9	4.9	10	-0.6	1	10	14.0%	7.50 [4.40 , 10.60]	-	+	+	+	!	-
**Total (95% CI)**			**139**			**136**	**100.0%**	**3.60 [1.74 , 5.47]**						

Heterogeneity: Tau² = 3.97; Chi² = 17.07, df = 7 (P = 0.02); I² = 59%
Test for overall effect: Z = 3.78 (P = 0.0002)
Test for subgroup differences: Not applicable

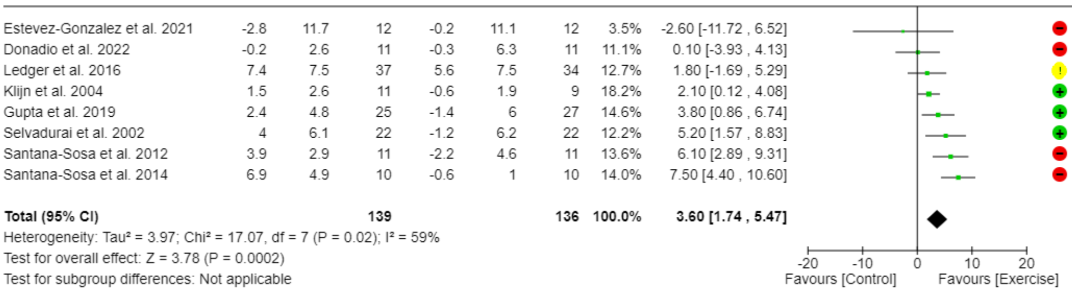

Risk of bias legend
(A) Randomisation process
(B) Deviations from the intended interventions
(C) Missing outcome data
(D) Measurement of the outcome
(E) Selection of the reported result
(F) Overall bias

+ Low risk
! Some concerns
- High risk

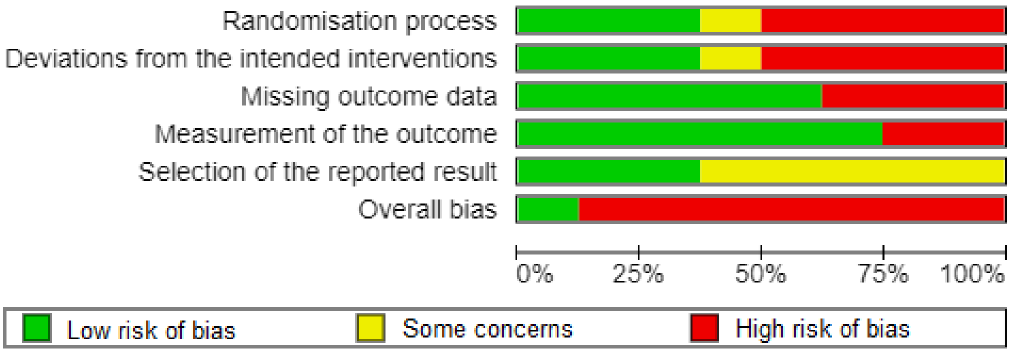

Figure S8. Physical activity level

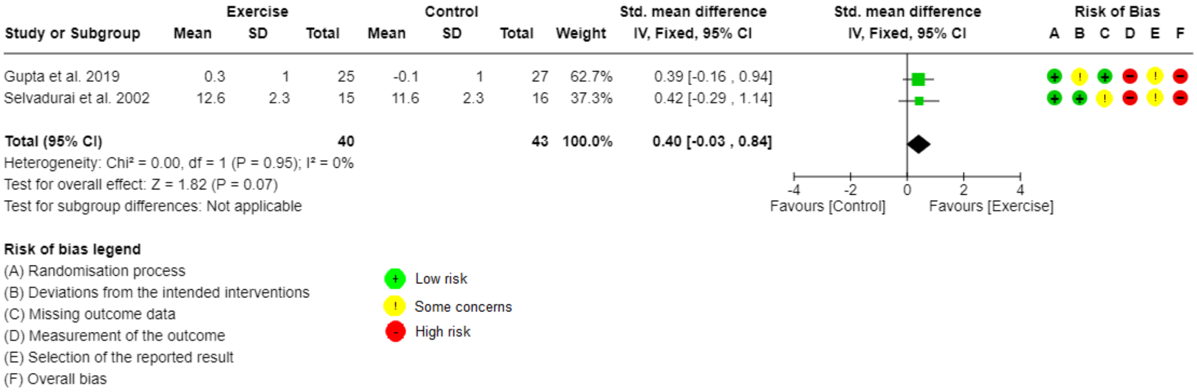

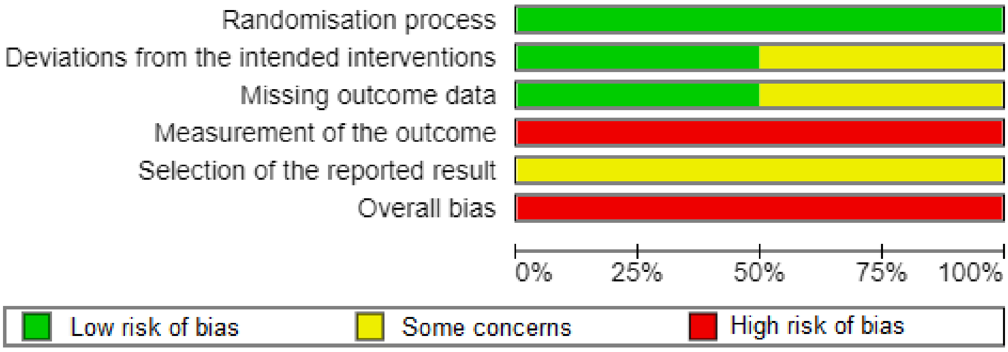

Supplement: Supplementary file 1 [file healthcare-10-02520-s001.zip › File S6 Secondary outcomes.pdf]
