# Supplementary material for: Effects of Exercise Training on Peripheral Muscle Strength in Children and Adolescents with Cystic Fibrosis: A Meta-Analysis
Source: Healthcare (Basel). 2022 Dec 13;10(12):2520. doi: 10.3390/healthcare10122520 (PMC9778003; doi:10.3390/healthcare10122520)

File S7. Summary of the risk of bias for the primary outcome

Figure S9. Lower limb muscle strength LM

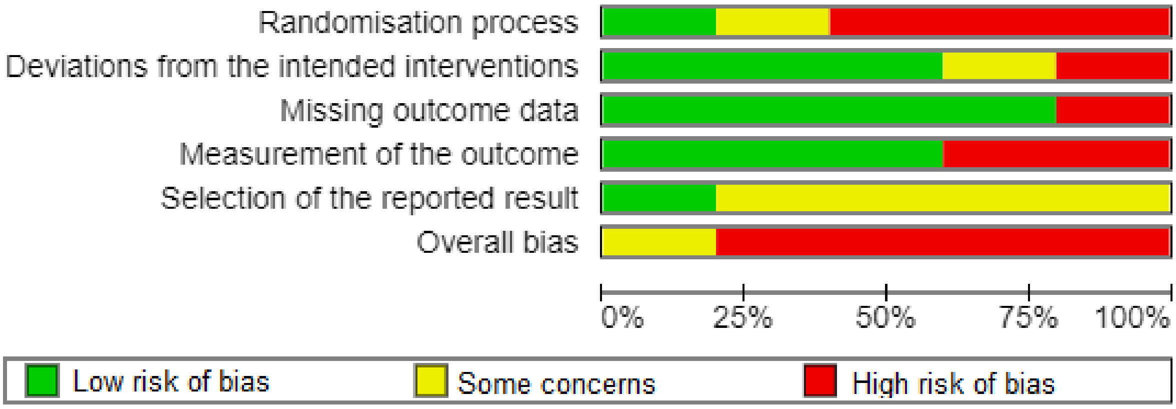

Figure S10. Upper limb muscle strength

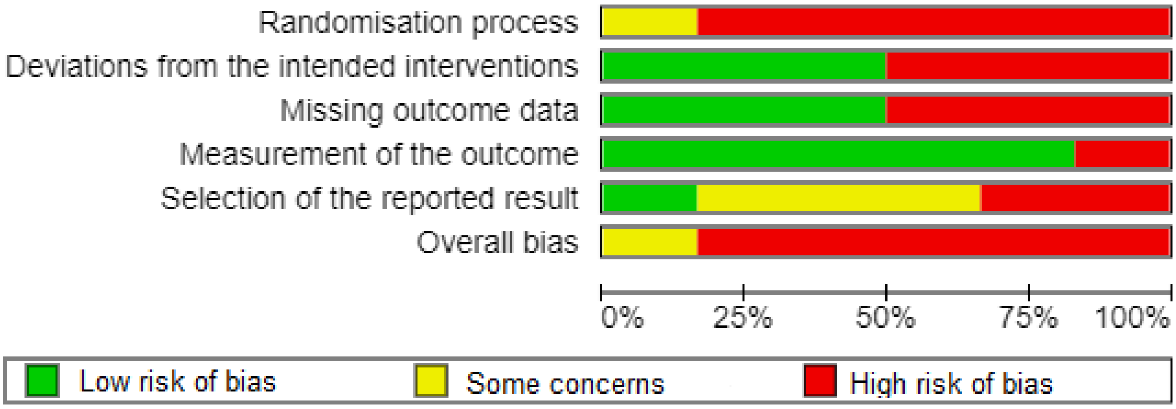

Supplement: Supplementary file 1 [file healthcare-10-02520-s001.zip › File S7 Summary of the risk of bias for the primary outcome.pdf]
